# Spectroscopic Evaluation of AlN/n-Si MIS Structures through Frequency-Driven Dielectric Characterization

**DOI:** 10.1021/acsomega.5c11218

**Published:** 2026-02-17

**Authors:** Abdullah Karaca, Dilber Esra Yıldız, Raziye Ertuğrul Uyar, Adem Tataroğlu

**Affiliations:** † Department of Physics, Faculty of Sciences, Yozgat Bozok University, Yozgat 66000, Turkey; ‡ Department of Physics, Faculty of Engineering and Natural Sciences, Hitit University, Corum 19030, Türkiye; § Department of Chemistry, Faculty of Arts and Sciences, Middle East Technical University, Ankara 06800, Turkey; ∥ Department of Physics, Faculty of Science, 37511 Gazi University, Ankara 06500, Turkey

## Abstract

This study presents a comprehensive spectroscopic and impedance-based analysis of a Au/Ti/AlN/n-Si metal–insulator–semiconductor (MIS) heterostructure, focusing on its frequency- and temperature-dependent electrical and dielectric behavior. The AlN interlayer, synthesized via hydride vapor-phase epitaxy (HVPE), was electrically characterized through admittance spectroscopy across a wide temperature range (100–350 K) and multiple frequencies (100, 500, and 1 MHz). Capacitance–voltage (*C–V*) and conductance–voltage (*G*/ω–*V*) measurements revealed strong dispersion effects, particularly at low frequencies and temperatures, where interfacial trap states and dipolar relaxation dominate the response. A negative capacitance behavior was observed under a reverse bias at low frequencies. Series resistance (*R*_s_) analysis confirmed a transition from trap-limited to bulk-controlled conduction with increasing frequency. Dielectric parameters, including real and imaginary permittivity, loss tangent, AC conductivity, and electric modulus components, exhibited a thermally activated and frequency-sensitive behavior, highlighting the interplay between dipolar alignment, trap reconfiguration, and energy dissipation. These findings provide critical insight into the interfacial physics of AlN/Si systems and establish a robust framework for optimizing AlN-based MIS devices for high-frequency, high-temperature microelectronic and optoelectronic applications.

## Graphical abstract

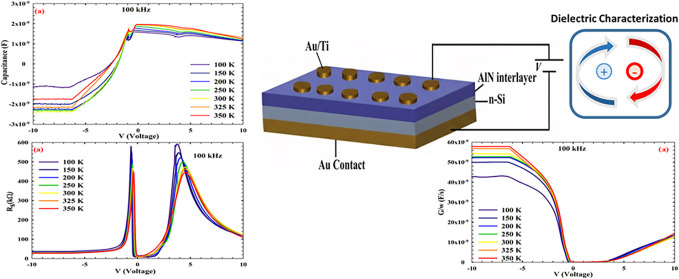

## 1 Introduction

The rapid evolution of modern electronic and photonic systems demands not only high-speed operation and energy efficiency but also resilience under extreme environmental conditions. As device architectures become increasingly complex, the role of interfacial engineering and dielectric stability has grown central to ensuring long-term reliability and performance. In particular, metal–insulator–semiconductor (MIS) or metal–oxide–semiconductor (MOS) and metal–insulator–metal (MIM) configurations have attracted significant attention due to their scalability and compatibility with integrated circuit platforms. These structures require dielectric materials that can maintain electrical integrity across a wide frequency spectrum, especially under thermal and electrical stress. Overall, the search for multifunctional thin films with a robust dielectric behavior has become a focal point in advanced materials research.^1–4^

Among the candidate materials, aluminum nitride (AlN) has emerged as a compelling choice due to its unique combination of properties: a wide direct band gap (∼6.2 eV), high thermal conductivity (∼340 W·m^–1^·K^–1^), excellent chemical inertness, and strong electrical insulation.^5–8^ These attributes make AlN suitable for both passive dielectric layers and active components in high-temperature and high-frequency applications. Its high piezoelectric coefficient and surface acoustic wave velocity further extend its utility to resonators, sensors, and ultraviolet photodetectors.^9–12^ Moreover, AlN’s compatibility with silicon substrates enables integration into existing CMOS technologies, although challenges such as lattice mismatch and interfacial defect formation remain critical concerns.^13^ Extensive research has been devoted to optimizing the growth techniques and structural quality of AlN thin films. Abdallah et al. investigated the thickness-dependent microstructural evolution of AlN layers deposited via reactive magnetron sputtering, revealing significant changes in grain morphology and crystallinity.^14^ Liu et al. performed a comparative analysis of AlN thin films deposited on different substrates, revealing that sapphire-based films exhibit larger grain sizes and increased surface roughness.^15^ In contrast, Kim et al. demonstrated that AlN films grown on silicon substrates provide enhanced optical transmittance and lower leakage currents. Furthermore, their study showed that the growth temperature in thermal ALD critically influences the crystallinity and resistive switching behavior of AlN.^16^ Mohan et al. analyzed the frequency-dependent capacitance and conductance characteristics of AlN/Si interfaces, emphasizing the role of metal contact selection in modulating interfacial trap dynamics.^17^ Pillai et al. developed solar-blind UV/IR photodiodes using AlGaN/Si heterostructures, achieving enhanced spectral selectivity through tunneling barrier engineering and doping optimization.^18^ Lu et al. addressed the intrinsic limitations of p-type doping in AlN by integrating p-Si nanomembranes with n-AlN layers, resulting in high-performance heterojunctions with improved carrier transport and voltage blocking capabilities.^19^ Kocyigit et al. demonstrated that AlN-based Schottky photodetectors fabricated via thermal evaporation exhibit high responsivity (1.36 A/W) and a fast transient response, confirming their suitability for visible light detection.^20^ Despite these advances, a comprehensive understanding of the interfacial behavior in AlN-based MIS structures remains incomplete. Capacitance–voltage (*C–V*) measurement and impedance spectroscopy have proven to be indispensable tools for probing interfacial phenomena, including the trap state density, series resistance, and dielectric loss mechanisms.^21–23^ These techniques enable the multidimensional evaluation of the electrical response, particularly under varying frequency and bias conditions. At low frequencies, the coupling between localized charge carriers and the oscillating electric field intensifies, manifesting as discernible deviations in the capacitance contour, indicative of trap-assisted polarization dynamics.^24,25^ The interplay between the frequency-dependent electrical response and interfacial dynamics necessitates a coupled analysis approach to accurately interpret the device behavior and predict long-term operational reliability.^26^ In our recent work, we fabricated and characterized an Au/Ti/AlN/n-Si structure using admittance spectroscopy to evaluate its dielectric and electrical response across a broad frequency and voltage range.^27^ The analysis revealed a pronounced frequency-dependent behavior in the capacitance (*C–V*), conductance (*G–V*), dielectric constant (*ε′*), loss factor (*ε″*), and electric modulus (*M′*, *M″*). Notably, interfacial trap states were found to dominate the low-frequency response, while high-frequency measurements exhibited reduced polarization due to limited charge mobility. Cole–Cole plots confirmed that grain boundary effects were more influential than bulk grain contributions in determining relaxation dynamics.

In this study, we present a comprehensive experimental investigation of a Au/Ti/AlN/n-Si heterostructure, focusing on its frequency- and temperature-dependent dielectric and electrical characteristics. The AlN layer was synthesized by using hydride vapor-phase epitaxy (HVPE) and subsequently integrated onto a silicon substrate to fabricate a MIS-type diode. Admittance spectroscopy served as the principal diagnostic tool to extract frequency- and temperature-resolved electrical parameters, including *C–V*, G–V, ε′, ε″, tan δ, *M′*, *M″*, and σ_ac_, over the 100–350 K range and at selected modulation frequencies of 100 kHz, 500 kHz, and 1 MHz. This targeted frequency regime enables high-resolution monitoring of interfacial trap dynamics and relaxation processes, particularly in domains in which carrier mobility and polarization exhibit pronounced frequency dependence. By correlating the thermal evolution of these electrical signatures with interfacial phenomena, the study establishes a multidimensional framework for assessing the operational reliability of AlN-based MIS structures. This study not only clarifies the interfacial trap dynamics in AlN/Si MIS structures but also establishes admittance spectroscopy as a predictive tool for device reliability in high-frequency applications. The findings contribute to a deeper understanding of AlN/Si interfaces and offer practical insights for the integration of AlN in high-frequency and temperature-dependent electronic and optoelectronic applications.

## 2 Experimental Section

The Au/Ti/AlN/n-Si heterostructure examined in this work was fabricated using a commercially sourced AlN-on-silicon template (MTI Corp.), featuring an n-type Si substrate with (111) orientation, 2 in. diameter, 0.5 mm thickness, and a resistivity of 1–10 Ω·cm. Prior to metallization, the wafer was sectioned into 1 × 2 cm^2^ units and subjected to sequential ultrasonic cleaning in 2-propanol, acetone, and deionized water, followed by nitrogen-assisted drying. Surface activation was enhanced via low-pressure plasma exposure (5 min), ensuring the removal of residual organics and improved adhesion. Ohmic contact on the rear Si surface was realized by thermal evaporation of a 100 nm Au layer under high vacuum (∼10^–6^ Torr), followed by annealing at 500 °C in ambient N_2_. The AlN dielectric, grown via hydride vapor-phase epitaxy (HVPE), exhibited a nominal thickness of 200 nm and served as the insulating interface. Schottky contacts were defined on the AlN surface using a patterned mask with 1 mm circular apertures, followed by the deposition of a Ti/Au bilayer (5/100 nm) via thermal evaporation. The Ti interlayer was selected to modulate the effective barrier height by leveraging its intermediate work function. The surface morphology of the AlN/n-Si samples was examined using a QUANTA 400F field emission scanning electron microscope operated at an accelerating voltage of 30 kV, a working distance of 12.7 mm, and a spot size setting of 3. Secondary electron micrographs were acquired at multiple magnifications to provide a multiscale assessment of the film integrity and morphology. These imaging conditions yield high-contrast, high-resolution views suitable for evaluating surface continuity, grain boundary structure, and substrate coverage. Electrical measurements were performed by using a Hewlett–Packard 4192A impedance analyzer. *C–V* and *G–V* profiles were acquired over a bias range of −10 to +10 V in 50 mV increments. Frequency-dependent admittance data were collected at 100 kHz, 500 kHz, and 1 MHz. Temperature-controlled measurements spanning 100–350 K were conducted using a Keithley 2400 source meter integrated with a Lake Shore 321 controller and a VPF-475 cryostat, ensuring thermal stability during spectroscopic interrogation.

Scheme 1a,b depicts the layered Au/Ti/AlN/n-Si MIS heterojunction and a qualitative equilibrium band alignment constructed from established material parameters. In this representation, the Au electrode is assigned a work function of ≈5.1 eV, the n-Si substrate an electron affinity of ≈4.05 eV, and the AlN interlayer a wide band gap of ≈6.2 eV.^28–30^ The thin Ti film is included primarily to promote adhesion and to improve metal–semiconductor contact formation, whereas the insulating AlN layer modifies the local electric field distribution and raises the effective barrier to carrier injection without supporting appreciable bulk conduction. The continuity of the electrochemical potential across the stack is shown schematically.^31,32^ The precise potential within AlN is strongly influenced by interface states, fixed charges, and possible Fermi level pinning at the metal/insulator and insulator/semiconductor boundaries. This band schematic provides a consistent physical framework for interpreting the observed electrical response. The effective barrier height controls the rectifying characteristics and turn-on behavior, whereas the presence of the AlN interlayer introduces interfacial regions where trap states and polarization effects can develop.^33–35^ Based on this configuration, AlN-based Schottky diodes were fabricated under optimized conditions and subjected to systematic electrical evaluation. In particular, the temperature dependence of capacitance and conductance was analyzed through frequency-resolved measurements, allowing the quantification of thermal activation processes and interfacial state behavior.

**Scheme 1:**
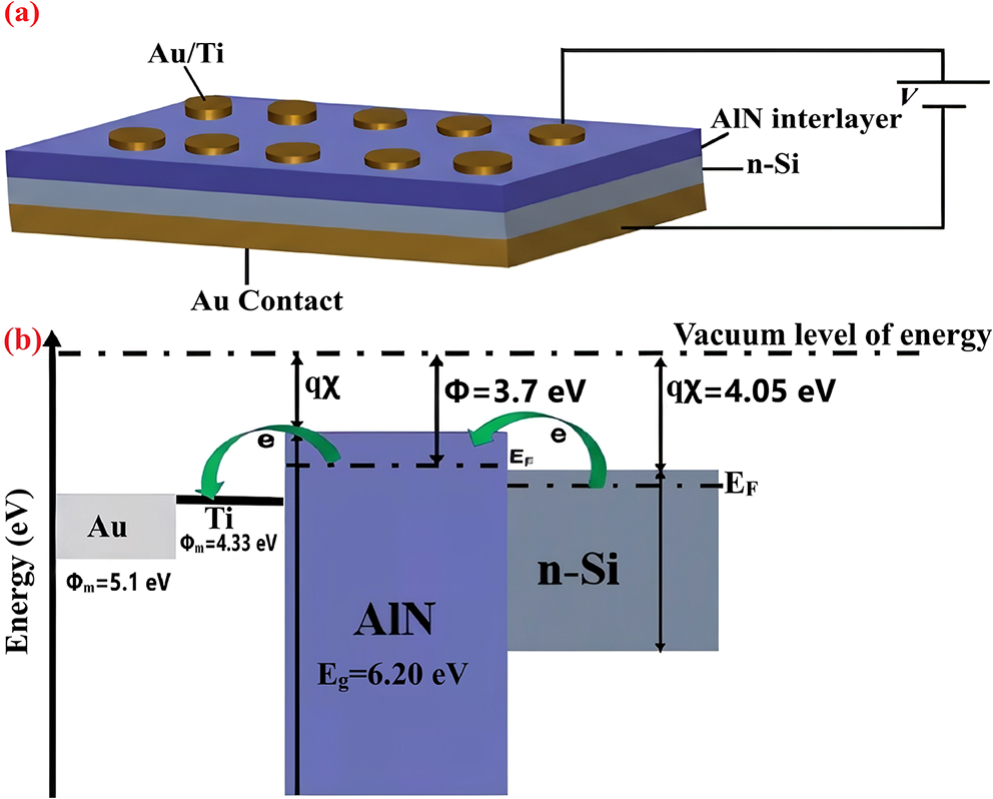
(a) Layered Architecture and (b) Equilibrium Energy-Band Profile of the Au/Ti/AlN/n-Si Metal–Insulator–Semiconductor (MIS) Heterojunction

## 3 Results and Discussion

Prior to the electrical and dielectric analyses, the surface morphology and structural integrity of the AlN/n-Si heterostructure were examined by scanning electron microscopy in order to assess the film uniformity and interface quality. Figure 1 displays SEM micrographs recorded at progressively increasing magnifications of ×1000, ×5000, ×10,000, and ×20,000, providing a multiscale view of the surface features. At low magnification (Figure 1a, ×1000), the AlN layer exhibits a continuous surface coverage over the n-Si substrate. The morphology is characterized by a cellular, porous-like network distributed relatively uniformly across the surface, suggesting that the deposition process yields a mechanically coherent film over large areas.^36–38^ Such continuity is essential for ensuring reproducible electrical contact formation in subsequent device fabrication steps. As the magnification increases to ×5000 (Figure 1b), the surface texture becomes more clearly resolved. The AlN film reveals interconnected regions separated by well-defined boundaries, forming micron-scale domains with irregular polygonal shapes.^39,40^ These features indicate a polycrystalline or nanostructured growth mode where coalescence of neighboring domains occurs during film formation. Importantly, no evidence of pinholes directly exposing the underlying silicon is observed at this scale, showing that the AlN layer effectively covers the substrate.^41,42^

**Figure 1:**
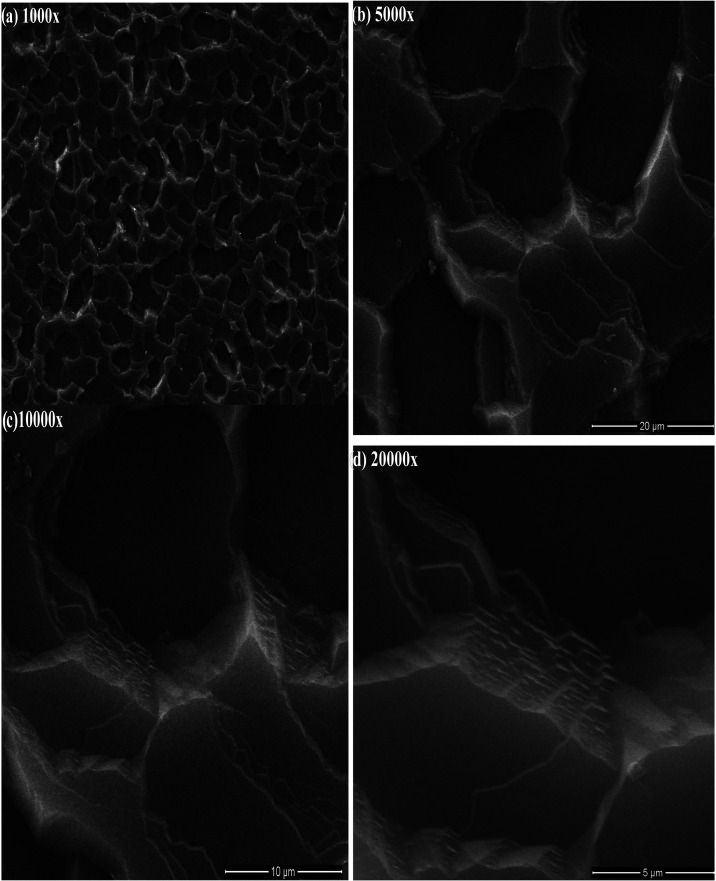
SEM images of the AlN/Si structure at different magnifications: (a) ×1000, (b) ×5000, (c) ×10,000, and (d) ×20,000.

Further enlargement to ×10,000 (Figure 1c) allows a closer inspection of the internal morphology of individual domains. The images show pronounced surface relief and localized thickness variations along the domain edges accompanied by fine surface undulations. Such features are commonly associated with strain relaxation and grain boundary formation in thin dielectric or nitride films.^43,44^ These microstructural characteristics can play a role in defining the local electric field distribution and interface state formation at the AlN/n-Si junction. At the highest magnification (Figure 1d, ×20,000), the granular nature of the AlN film becomes evident. Submicron-scale features and layered contrasts are visible along the domain boundaries, suggesting a stacked or columnar growth tendency. While the surface is not atomically flat, the absence of large voids or discontinuities indicates that the film maintains structural integrity at the nanoscale.^45^ This morphology is consistent with a dense yet textured dielectric layer, which may contribute to the interface-related electrical responses observed in frequency-dependent measurements. Overall, the SEM analysis confirms that the AlN layer forms a continuous, structurally stable film on n-Si, with a hierarchical morphology ranging from micrometer-scale domains to nanoscale granular features.

### 3.1 Frequency- and Temperature-Dependent Capacitance Behavior

The capacitance–voltage (*C–V*) behavior of the Au/Ti/AlN/n-Si MIS structure was systematically examined across three distinct frequencies, 100, 500, and 1000 kHz, within a temperature range spanning 100 to 350 K. The resulting data, as presented in Figure 2, reveal a pronounced dependence of the dielectric response on both thermal activation and signal frequency. At 100 kHz (Figure 2a), the *C–V* profiles exhibit steep transitions near zero bias, particularly at low temperatures (100–200 K), where the curves are markedly asymmetric. This behavior is indicative of frozen interface states and limited carrier mobility, which restrict the dynamic response of the depletion region. As the temperature increases, the curves broaden and become more symmetric, reflecting the thermal activation of trap states and enhanced carrier transport. The observed shift in peak capacitance with temperature suggests a redistribution of interfacial charges and the onset of dipolar relaxation phenomena. Comparable trends have been reported in AlN-based MIS structures, where low-frequency measurements are dominated by interface trap contributions that follow the AC signal and augment the measured capacitance.^27,46^

**Figure 2:**
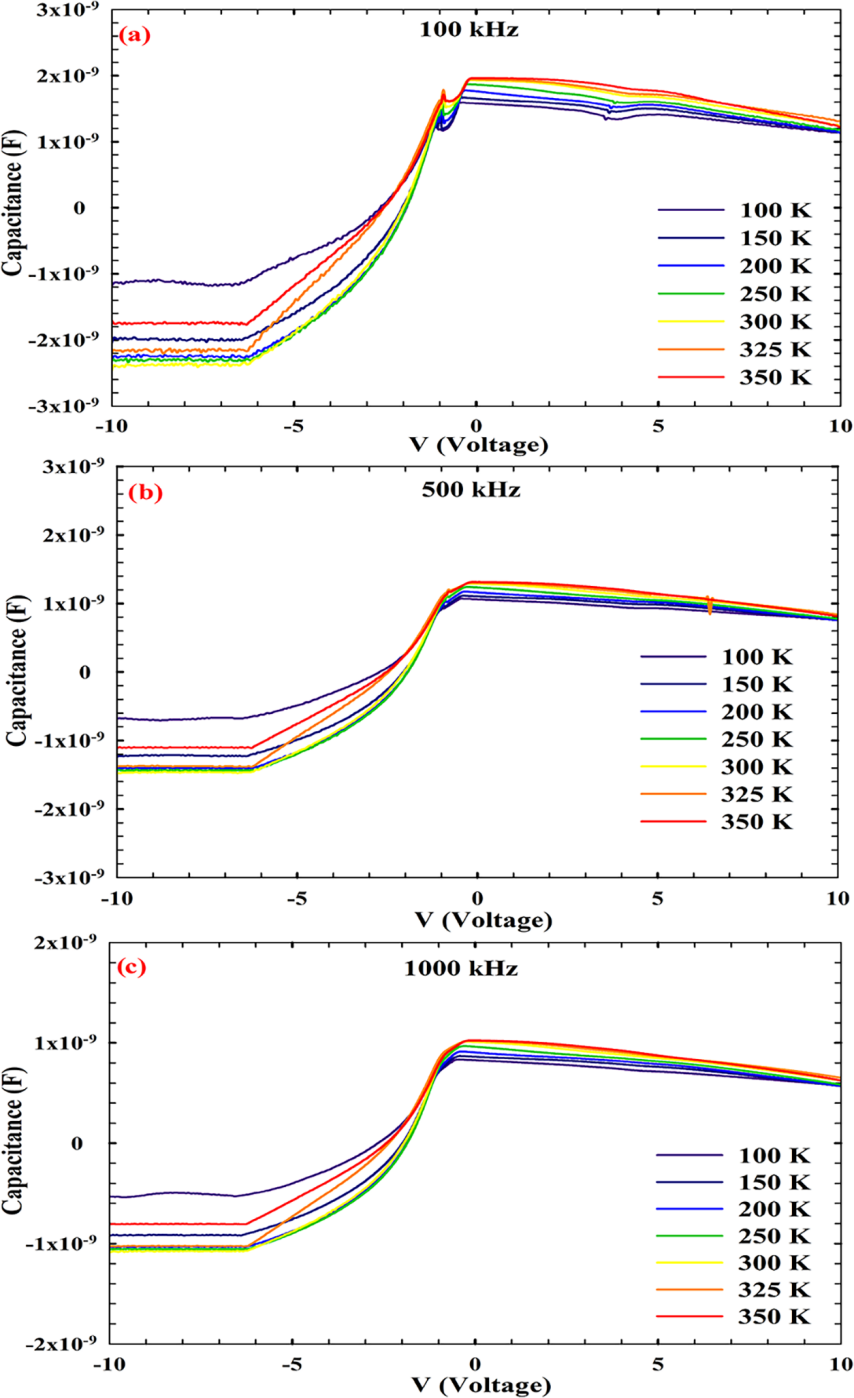
*C–V* response of the AlN/n-Si MIS structure measured in the 100–350 K temperature range at different frequencies: (a) 100, (b) 500, and (c) 1000 kHz.

At 500 kHz (Figure 2b), the capacitance curves display improved symmetry and elevated peak amplitudes with an increasing temperature. This frequency regime suppresses the response of slower trap states, thereby accentuating the intrinsic dielectric behavior of the AlN layer. The temperature-induced increase in capacitance is attributed to enhanced dipole alignment and reduced series resistance. A similar frequency-dependent suppression of trap-related effects has been documented in SnO_2_-based MIS devices, where deep-level traps fail to respond at elevated frequencies, resulting in stabilized capacitance values.^47^At 1000 kHz (Figure 2c), the *C–V* characteristics exhibit minimal distortion and a well-defined plateau, particularly at higher temperatures (300–350 K). This regime effectively isolates the bulk dielectric response of the AlN layer with negligible contribution from interfacial defects. The overall evolution of the *C–V* profiles across the examined frequency and temperature domains shows the interplay between trap dynamics, dielectric relaxation, and carrier mobility. A distinct anomaly is observed in the reverse bias region at 100 kHz, where the capacitance approaches zero and, under certain conditions, becomes negative. This deviation from the ideal depletion behavior is symptomatic of delayed carrier transport and dynamic charge redistribution. Negative capacitance (NC) under reverse bias has been previously reported in p–i–n diodes,^48^ III–V Schottky structures,^49^ and metal–semiconductor–metal (MSM) structures.^50^ Kumar et al. demonstrated that in thin-film organic homojunctions, reverse bias capacitance can fall below the geometrical limit due to a time lag between carrier injection and charge storage.^51^ When the probing frequency is lower than the carrier transit time, the differential charge response becomes negative, yielding a net decrease in the stored charge with increasing bias. This phenomenon can be described by the small-signal capacitance definition: *C* = *dQ*/*dV*. Under NC conditions, *dQ*/*dV* < 0, indicating that an increase in bias voltage leads to a reduction in net charge. In the present AlN/Si MIS structure, the emergence of NC is attributed to trap-assisted carrier dynamics, interfacial polarization, and series resistance effects. At low frequencies, interface states are capable of tracking the AC signal; however, under reverse bias, their delayed discharge leads to a reduction in net charge, manifesting as a negative capacitance component. This behavior is inherently frequency-dependent and diminishes at higher frequencies, where trap states are unable to respond within the signal period. The observed behavior aligns with admittance spectroscopy models, wherein the low-frequency capacitance is strongly modulated by the interface state density (*D*_it_), while high-frequency measurements predominantly reflect bulk dielectric properties. Hoffmann et al. emphasized that in ferroelectric and high-k dielectrics such as HfO_2_, frequency-dependent permittivity and polarization dynamics are critical for understanding energy dissipation and charge transport mechanisms.^52^ The temperature-dependent evolution of the *C–V* profiles shown in Figure 2 thus provides insight into the coupled effects of interfacial phenomena and the intrinsic dielectric behavior in AlN-based MIS structures.

The *G*/ω*–V* conductance profiles of the Au/Ti/AlN/n-Si MIS structure, measured at 100, 500, and 1000 kHz across a temperature range of 100–350 K, reveal a frequency-governed transition in the dominant transport mechanisms, as depicted in Figure 3. At low frequencies and cryogenic temperatures, the conductance remains elevated across a wide bias range, suggesting that slow trap states and dipolar entities are actively contributing to the AC response. These entities can follow low-frequency excitation, leading to enhanced dielectric loss and elevated *G*/ω values, particularly under reverse bias conditions. As the excitation frequency increases, a systematic suppression of the conductance is observed. This attenuation is attributed to the inability of sluggish interfacial processes to respond within a shortened signal period. At intermediate frequencies, the conductance profiles begin to flatten and the bias-dependent asymmetry diminishes, indicating a transition from trap-dominated to bulk-controlled transport. The temperature sensitivity also weakens, consistent with the reduced participation of the thermally activated traps. At high frequencies, the *G*/ω curves converge across all temperatures and bias conditions, revealing a regime dominated by fast carrier dynamics and intrinsic dielectric behavior. At higher frequencies, the conductance approaches a stable value, indicating that contributions from interfacial polarization and trap-related relaxation are significantly reduced. This convergence confirms that the measured *G*/ω*–V* values at elevated frequencies predominantly reflect the intrinsic loss mechanisms of the AlN dielectric and the mobility of the majority of carriers within the semiconductor substrate. This frequency-dependent evolution of conductance can be interpreted within the framework of admittance spectroscopy, where the total conductance is expressed as^53^

1 $$G\left(\omega \right)=\omega C_{\mathrm{eff}}\;\tan\delta$$

Here, *C*_eff_ denotes the effective capacitance and tan δ represents the dielectric loss tangent, which is strongly influenced by trap relaxation and interfacial polarization. At low frequencies, tan δ is elevated due to slow trap dynamics, resulting in higher *G*/ω values. As the frequency increases, these contributions diminish and the conductance reflects the intrinsic dielectric loss of the AlN layer. A distinct anomaly emerges under reverse bias at low frequencies, where the capacitance approaches zero and, under certain conditions, becomes negative. Kumar et al. demonstrated that in thin-film homojunction diodes, reverse bias capacitance can fall below the geometrical limit due to field-dependent mobility and time-lagged carrier injection.^51^ When the probing frequency is lower than the carrier transit time, the differential charge response becomes negative:

2 $$C=\frac{\mathrm{dQ}}{\mathrm{dV}}<0$$

This behavior was modeled using a phenomenological expression incorporating Fowler–Nordheim tunneling and Poole–Frenkel-type mobility modulation:^54^

3 $$C=C_{g}-\frac{\mathrm{AV}}{d}\;\exp\left(-\alpha V-\beta \sqrt{V}\right)$$

The negative term arises from the delay between carrier injection and charge storage, which becomes significant at low frequencies. The comparative analysis across the three frequencies reveals a consistent trend; increasing the measurement frequency progressively attenuates the influence of slow-responding mechanisms, such as interface traps and deep-level defects. While low-frequency measurements are sensitive to defect-related phenomena and interfacial quality, high-frequency data provide a more accurate representation of the fundamental electrical properties of the MIS structure. However, to fully interpret the electrical response, particularly under low-frequency and low-temperature conditions, it is essential to account for the influence of series resistance (*R*_s_), which can significantly distort both conductance and capacitance measurements.

**Figure 3:**
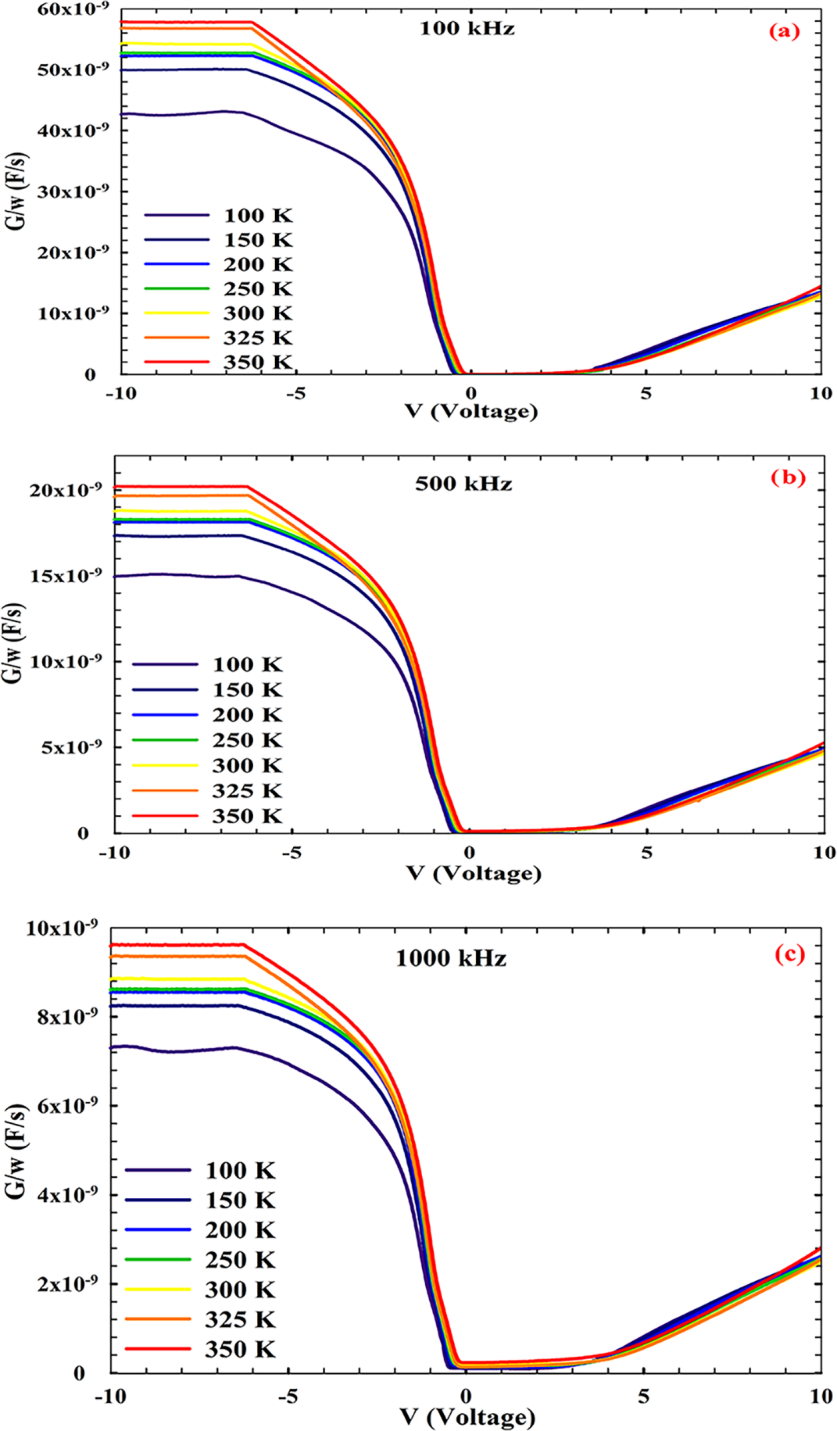
*G*/ω–*V* response of the AlN/n-Si MIS structure measured in the 100–350 K temperature range at different frequencies: (a) 100, (b) 500, and (c) 1000 kHz.

The *R*_s_ values were calculated using the Nicollian–Brews formalism, which relates the measured capacitance (*C*_m_) and conductance (*G*_m_) to the intrinsic device parameters through the following expression:^55^

4 $$R_{s}=\frac{G_{m}}{G_{m}^{2}+({\omega C_{m})}^{2}}$$

This formulation assumes a parallel *RC* equivalent circuit and isolates *R*_s_ from the total impedance by accounting for both capacitive and conductive contributions. The frequency-dependent term ω*C*_m_ ensures that *R*_s_ extraction remains valid under varying signal conditions. The *R*_s_–V profiles of the AlN-based MIS structure, recorded across three distinct frequencies (100, 500, and 1000 kHz), demonstrate a pronounced interplay between thermal activation and frequency-dependent carrier dynamics, as illustrated in Figure 4. At 100 kHz, the elevated *R*_s_ values observed at cryogenic temperatures (100–200 K) suggest a conduction regime dominated by limited carrier mobility and deep-level interface traps. In this regime, reduced thermal excitation hinders trap ionization, leading to enhanced recombination and increased resistive losses. This behavior is consistent with prior reports on wide-band-gap MIS structures, where low-frequency measurements accentuate trap-mediated conduction pathways.^56,57^ As the frequency increases to 500 kHz, a systematic decline in *R*_s_ across the voltage range is observed, indicating that trap-mediated recombination is partially suppressed. The probing frequency likely exceeds the response time of slow traps, thereby reducing the contribution of cations to carrier capture and release. This transition reflects a shift toward a more intrinsic dielectric behavior of the AlN layer, where capacitive coupling begins to dominate resistive dissipation. Similar trends have been reported in Al_2_O_3_- and Si_3_N_4_-based MIS systems, where frequency-dependent impedance spectroscopy reveals trap deactivation above critical modulation rates.^58,59^ At 1000 kHz, the series resistance remains consistently low even at elevated temperatures, suggesting that rapid field oscillations inhibit trap reconfiguration and facilitate swift carrier transitions across the MIS interface. The observed behavior shows the dielectric responsiveness of the AlN interlayer and its coupling to the carrier concentration in the n-type Si substrate. These results confirm the suitability of AlN-based MIS interfaces,particularly for high-frequency electronic applications requiring interface stability, low-loss operation, and dynamic load modulation.

**Figure 4:**
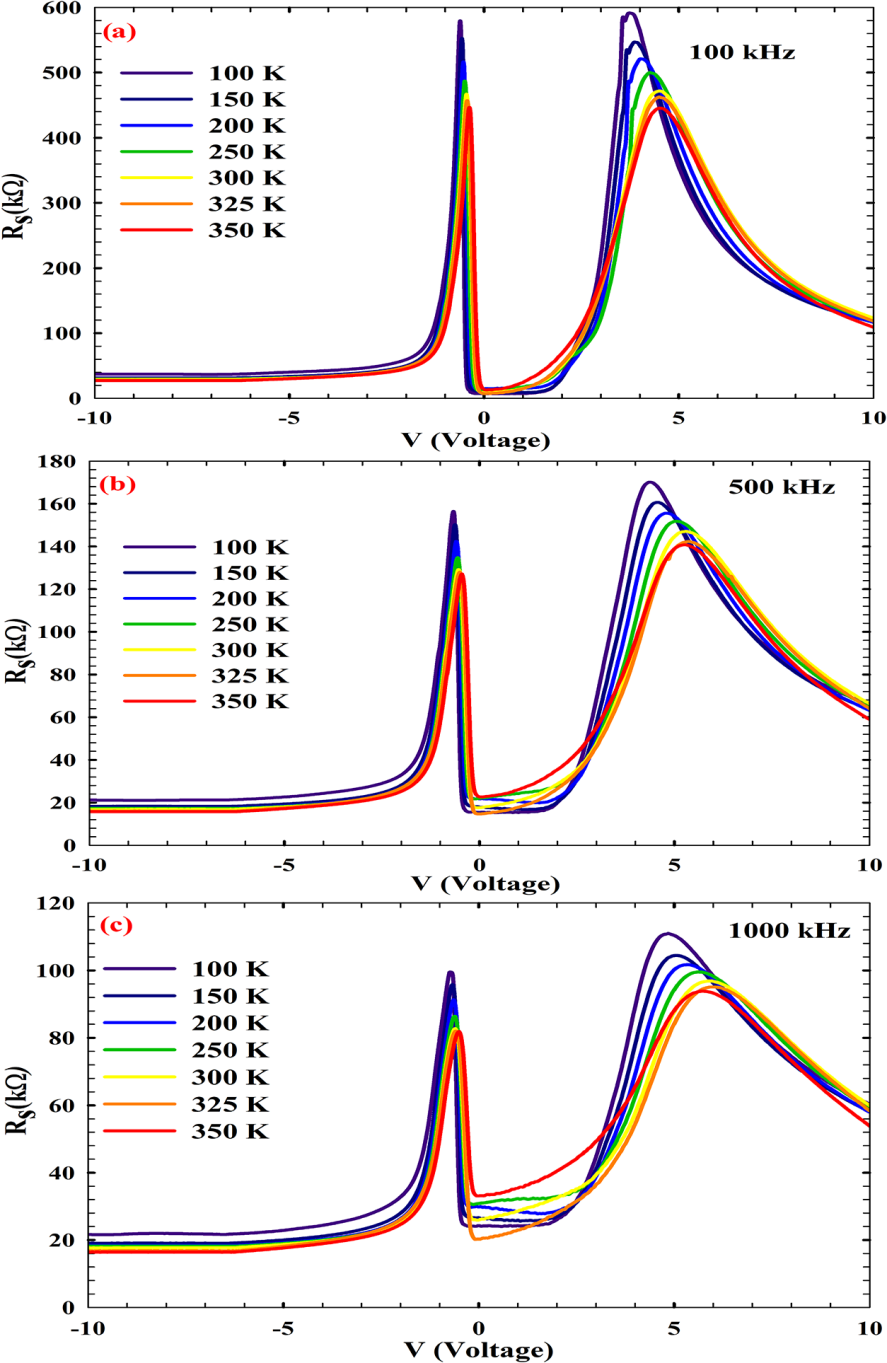
Bias-dependent *R*_s_ profiles of AlN/n-Si MIS at (a) 100, (b) 500, and (c) 1000 kHz.

While Figure 4 elucidates the frequency-dependent evolution of the series resistance (*R*_s_) under varying thermal conditions, Figure 5 extends this analysis by probing the dielectric and conductive response of the AlN-based MIS structure through temperature-dependent capacitance (*C*) and conductance (*G*/ω) measurements. Both parameters exhibit a monotonic increase with temperature, reflecting enhanced dipolar polarization and a thermally activated carrier response. At 100 kHz, the elevated *C* and *G*/ω values suggest that low-frequency probing allows sufficient time for interfacial dipoles and trap states to respond to the applied field, resulting in higher dielectric storage and loss. The convergence of *C* and *G*/ω values at high temperatures and high frequencies points to a regime where the intrinsic dielectric behavior dominates over interfacial contributions.^60^ This transition from resistive to reactive parameters marks a shift in focus, from carrier transport limitations to dielectric polarization and interfacial dynamics. At 100 kHz, both *R*_s_ (Figure 4a) and *C* (Figure 5a) exhibit elevated values at low temperatures, indicative of trap-limited conduction and enhanced dipolar alignment within the AlN layer. As the frequency increases to 500 and 1000 kHz, the decline in *R*_s_ is mirrored by a systematic reduction in *C* and *G*/ω, suggesting that high-frequency field oscillations suppress trap reconfiguration and limit dipole relaxation. Importantly, the parallel trends in *R*_s_, *C*, and *G*/ω across the temperature spectrum reinforce the notion that interfacial trap states and dielectric relaxation mechanisms are thermally activated and frequency-sensitive. The convergence of low *R*_s_ and suppressed *G*/ω at 1000 kHz highlights the onset of capacitive dominance, where the AlN layer behaves as a stable high-frequency dielectric with minimal loss. This validates the structural integrity and electronic suitability of the AlN/Si interface for RF and GHz-range applications, where a low-loss, thermally stable dielectric response is essential.

**Figure 5:**
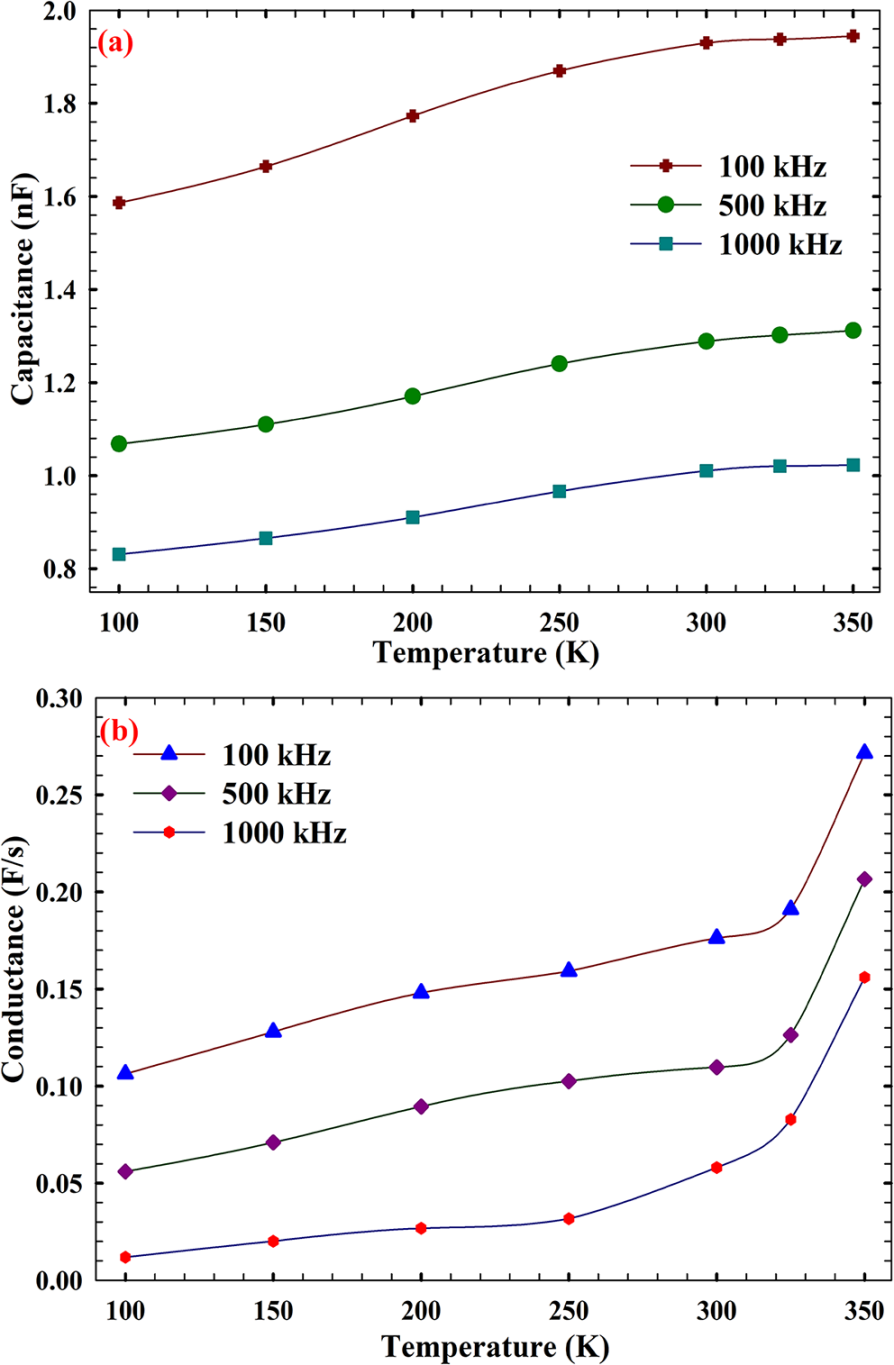
(a) Capacitance (*C*) and (b) conductance (*G*/ω) temperature dependence for the AlN/n-Si MIS structure at selected frequencies.

### 3.2 Dielectric Response Analysis of the AlN/n-Si MIS Structure

The dielectric characterization of AlN-based metal–insulator–semiconductor (MIS) structures was conducted using the complex permittivity formalism expressed as^61^

5 $$\varepsilon {\;}^{*}=\varepsilon^{\prime }-{j\varepsilon }^{\prime \prime }$$

Here, ε′ denotes the real part of the permittivity, representing the material’s ability to store electrical energy under an applied field, primarily governed by dipolar and space charge polarization mechanisms. The imaginary component, ε*″*, accounts for energy dissipation within the dielectric medium arising from relaxation losses, interfacial trap dynamics, and thermally activated conduction. The dielectric loss factor, tan δ, quantifies the ratio of energy lost to energy stored per cycle and is defined as^62,63^

6 $$\tan\delta =\frac{\varepsilon^{\prime \prime }}{\varepsilon^{\prime }}$$

In addition, the AC conductivity (σ_ac_) provides insight into the frequency-dependent charge transport behavior and is calculated via^64,65^

7 $$\sigma_{\mathrm{ac}}=\varepsilon_{0}\varepsilon^{\prime \prime }\omega$$

where ε_0_ is the vacuum permittivity (8.854 × 10^–12^F/m), and ω = 2πf is the angular frequency corresponding to the applied signal. These dielectric parameters, ε′, ε″, tan δ, and σ_ac_, serve as critical indicators of the AlN layer’s polarization dynamics, trap-mediated relaxation processes, and thermally modulated carrier mobility. Their dependence on both the temperature and the frequency enables a comprehensive understanding of interfacial stability, dipolar responsiveness, and energy dissipation mechanisms within the MIS architecture.

The dielectric behavior of the AlN/n-Si MIS structure was systematically evaluated through temperature-dependent measurements of real permittivity (ε′), imaginary permittivity (ε*″*), loss tangent (tan δ), and AC conductivity (σ_ac_), across three probing frequencies: 100, 500, and 1000 kHz. These parameters, as presented in Figure 6a–d, provide a comprehensive view of the polarization dynamics, energy dissipation mechanisms, and thermally activated conduction processes within the AlN interlayer. As the temperature increases, the real part of the permittivity (ε′) exhibits a systematic rise across all measured frequencies, reflecting enhanced dipolar alignment and thermally activated space charge polarization within the AlN/n-Si MIS structure. This behavior is particularly pronounced at 100 kHz, where ε′ reaches a maximum of 6.07 at 350 K, indicating that low-frequency fields allow sufficient time for dipoles and interfacial charges to respond to the applied stimulus. The observed frequency-dependent attenuation in ε*′*, with values decreasing progressively at 500 and 1000 kHz, confirms the dispersive nature of the dielectric response, consistent with Debye-type relaxation and the limited ability of slower polarization mechanisms to follow rapid field oscillations.^66^ In parallel, the imaginary permittivity (ε″), which quantifies dielectric losses due to relaxation and conduction processes, also increases with the temperature, rising from 0.37 to 0.67 at 100 kHz. This trend suggests that thermally activated carriers and interfacial trap states contribute significantly to energy dissipation, particularly under low-frequency excitation where trap reconfiguration is more pronounced. At higher frequencies, the suppression of ε″ indicates that fast field modulation effectively inhibits trap dynamics, thereby reducing loss contributions. This frequency-dependent damping is further corroborated by the loss tangent (tan δ) profiles, which peak at 0.110 (100 kHz, 350 K), highlighting the dominance of interfacial relaxation and trap-assisted conduction under thermally favorable conditions.^67,68^

**Figure 6:**
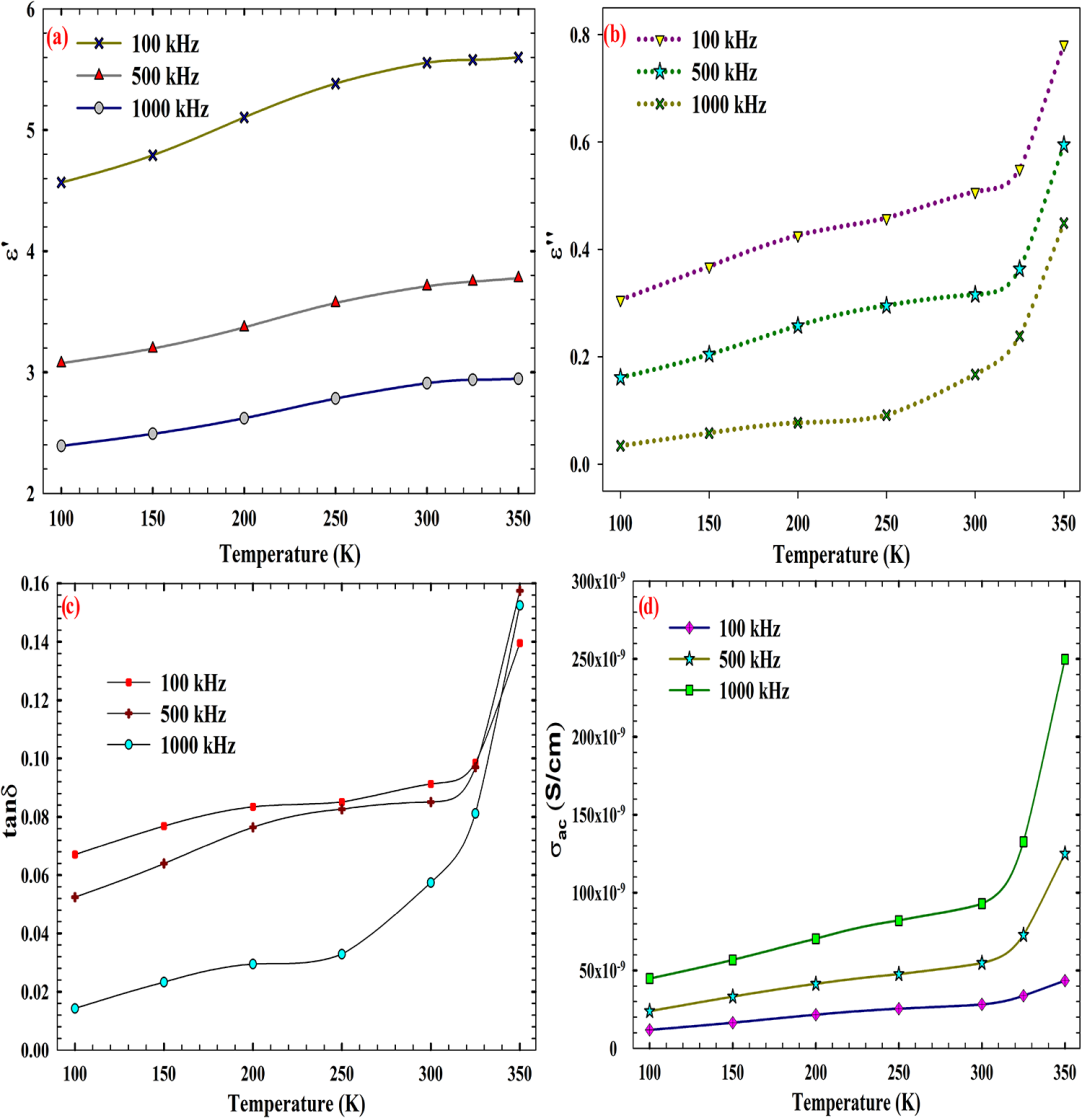
Thermal evolution of (a) ε′, (b) ε″, (c) tan δ, and (d) σ_ac_ in the AlN/n-Si MIS structure across selected frequencies.

The AC conductivity (σ_ac_), derived from ε″ and the angular frequency, reveals a strong temperature dependence, increasing steadily across all frequencies. Notably, the 500 kHz data exhibit the highest conductivity values, reaching 1.25 × 10^–7^ S/cm at 350 K. This suggests an optimal interplay between trap activation and carrier mobility at intermediate frequencies, where both capacitive and conductive mechanisms contribute synergistically to charge transport. In contrast, the lower σ_ac_ values observed at 1000 kHz reflect a regime dominated by capacitive behavior, with minimal trap participation and suppressed hopping conduction, consistent with the trends observed in ε′ and tan δ. These findings collectively show the frequency-adaptive dielectric behavior of the AlN layer, highlighting its potential for integration into high-frequency, low-dissipation electronic platforms, where interfacial robustness and agile charge dynamics are essential.

To deepen the understanding of charge transport mechanisms in the AlN/n-Si MIS structure, Figures 7 and 8 investigate the temperature-dependent conductivity behavior using two complementary models: the Arrhenius relation and the universal power law. As shown in Figure 7, the variation of ln­(σ) versus 1000/*T* across three frequencies (100, 500, and 1000 kHz) reveals a clear linear trend, confirming that conduction is thermally activated. The governing relation^69^

8 $$\sigma \left(T\right)=\sigma_{0}\;\exp\left(-E_{a}/\mathrm{kT}\right)$$

describes the exponential dependence of conductivity on temperature, where *E*_a_ is the activation energy and *k* is Boltzmann’s constant.^70^ Extracted activation energies (*E*_a_) and pre-exponential conductivity factors (σ_0_) for the AlN/n-Si MIS structure across two thermal regions—Region 1 (100–250 K) and Region 2 (300–350 K)—at three frequencies (100, 500, and 1000 kHz), based on Arrhenius fitting of the temperature-dependent conductivity data, are given in Table 1. The slopes of the ln­(σ) plots, as detailed in Table 1, indicate that the activation energy decreases with increasing frequency below room temperature and increases above room temperature. This change occurs as a result of electron hopping between localized states.^71,72^ In the variation of *E*_a_ with frequency, for example, at 100 kHz, the steep slope corresponds to deeper trap involvement and higher energy barriers, whereas at 1000 kHz, the flatter slope reflects reduced activation thresholds and enhanced carrier mobility under rapid field oscillations. This frequency-dependent modulation of *E*_a_ suggests a transition from trap-assisted hopping to more delocalized transport pathways as the probing frequency increases. In addition, for two regions, both *E*_a_ and σ_0_ values increase with the increase of temperature.

**Figure 7:**
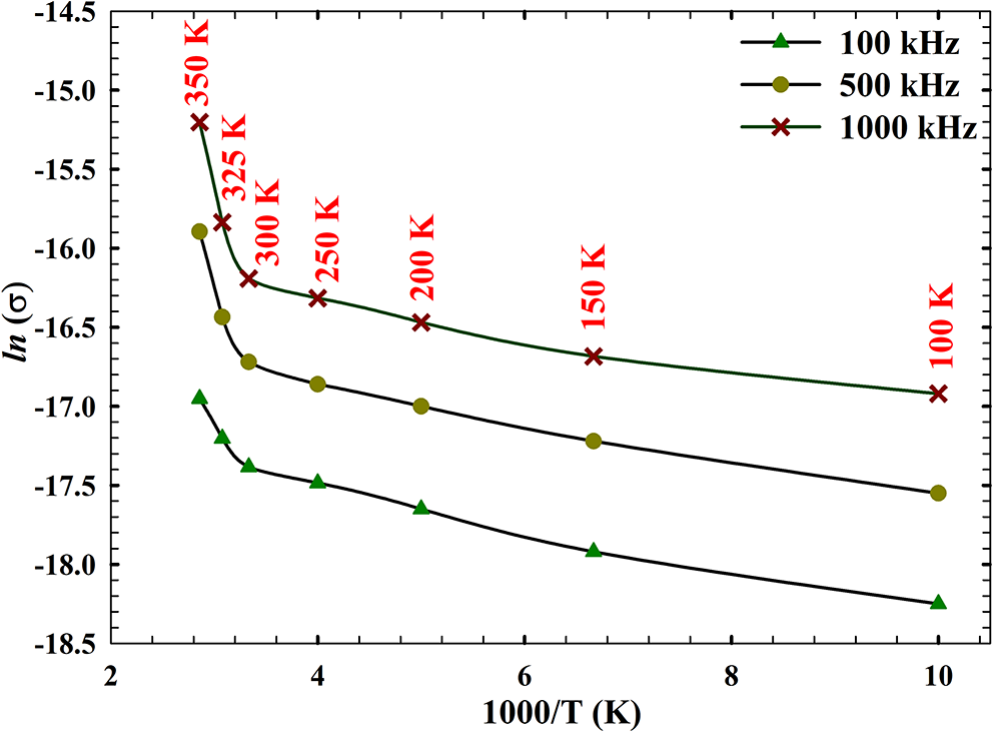
Variation of ln­(σ) versus 1000/*T* across three frequencies.

**Figure 8:**
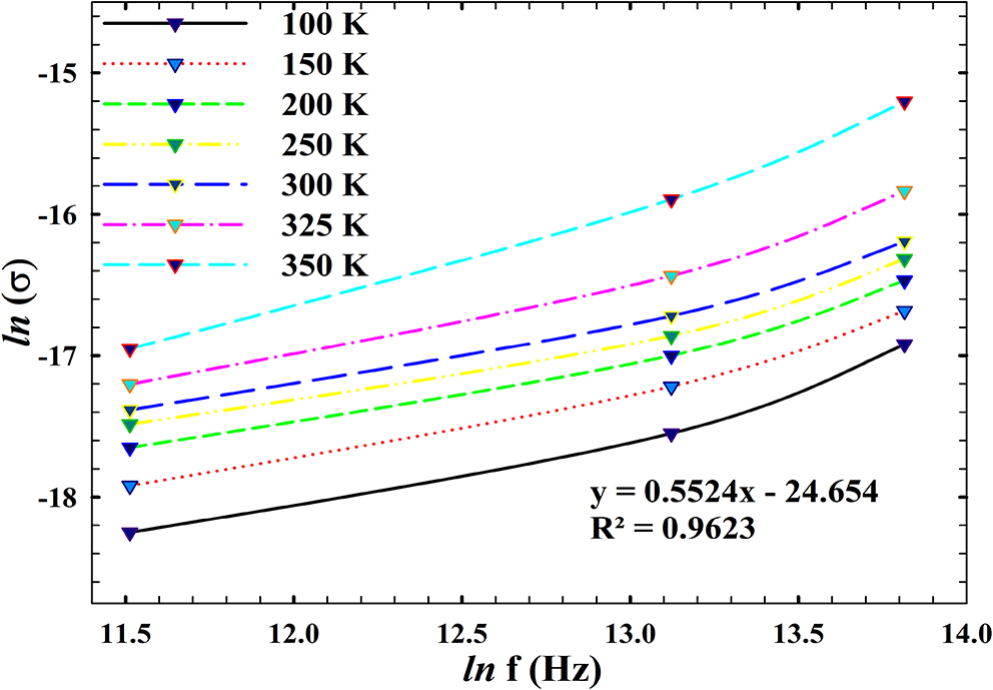
Frequency dependence of conductivity at fixed temperatures using the universal power law relation.

**Table 1:** Frequency-Dependent Activation Energies (*E*_a_) Extracted from Arrhenius Fitting for Two Thermal Regions

	**region 1** (100–**250 K)**			**region 2** (300–**350 K)**	
***f* (Hz)**	***E***_**a**_ (**meV**)	**σ**_0_	***f* (Hz)**	***E***_**a**_ (**meV**)	**σ**_0_
1,00,000	10.87	4.07 *×* 10^–8^	1,00,000	77.83	5.62 × 10^–7^
5,00,000	9.82	7.35 × 10^–8^	5,00,000	147.88	1.58 × 10^–5^
10,00,000	8.48	1.16 × 10^–7^	10,00,000	177.44	8.37 × 10^–5^

The frequency dependence of conductivity at fixed temperatures was analyzed by the universal power law.^73–75^

9 $$\sigma_{\mathrm{ac}}=\sigma_{\mathrm{dc}}+A\omega^{s}$$

Here, “*s*” is the frequency exponent that can vary in the value range 0 < *s* < 1. The ln­(σ) versus ln­(*f*) plots are illustrated in Figure 8. The “*s*” values were determined from the slope of the linear parts of these plots and are given in Table 2. The “*s*” value decreases at temperatures below room temperature and increases above room temperature. These plots reveal that the frequency exponent “*s*” varies systematically with temperature, offering insight into the dominant conduction mechanism. At lower temperatures, elevated “*s*” values are indicative of localized hopping and a strong trap influence, consistent with polaronic transport or tunneling through defect states. As the temperature rises, the exponent “*s*” decreases, reflecting a shift toward extended-state conduction where carriers interact less with localized traps and more with the bulk band structure.

**Table 2:** Variation of *s* and *W*_m_ with Temperature

***T***	***s***	***W***_**m**_ **(meV)**
100	0.55	115.62
150	0.52	161.38
200	0.49	204.42
250	0.48	251.16
300	0.50	310.07
325	0.57	393.97
350	0.74	700.14

Additionally, the temperature dependence of “*s*” was analyzed with the correlated barrier hopping (CBH) model. According to this model, *W*_m_ describes the binding energy of the charge carrier in localized sites. *W*_m_ is also called the “maximum barrier height”. The magnitude of *W*_m_ is computed from the following equation:^47,48^

10 $$s=1-\frac{6k_{B}T}{W_{m}}$$

Calculated binding energy *W*_m_ values are given in Table 2. The *W*_m_ value increases with an increasing temperature. This increase is due to the increase in disorder that occurs with increasing temperature. Furthermore, the temperature dependence of the frequency exponent (*s*) is consistent with the CBH model, especially at temperatures below room temperature.

### 3.3 Electric Modulus Formalism and Relaxation Dynamics

Building upon the insights gained from conductivity and dielectric loss behavior, the subsequent analysis in Figure 9 adopts the electric modulus formalism to further probe the relaxation dynamics within the AlN/n-Si MIS structure. Unlike permittivity-based approaches, which can be dominated by electrode polarization effects at low frequencies, the modulus representation emphasizes the bulk material response and is particularly effective in isolating localized relaxation phenomena. This framework enables a more refined interpretation of how dipolar entities and trap states evolve under varying thermal and electrical conditions. Figure 9a,b presents the temperature-dependent variation of the imaginary components of the electric modulus (*M′* and *M″*) across three frequencies (100, 500, and 1000 kHz). These quantities are derived from the inverse of complex permittivity^76,77^

11 $$M^{*}=\frac{1}{\varepsilon^{*}}=M^{\prime }+jM^{\prime \prime }$$

where *M′* reflects the out-of-phase component associated with energy dissipation during relaxation, and *M″* serves as a higher-order derivative sensitive to subtle transitions and nonlinear dielectric behavior. The evolution of these parameters with temperature and frequency provides critical insight into the relaxation time distribution, interfacial dynamics, and degree of coupling between localized states and the external field.^78^

**Figure 9:**
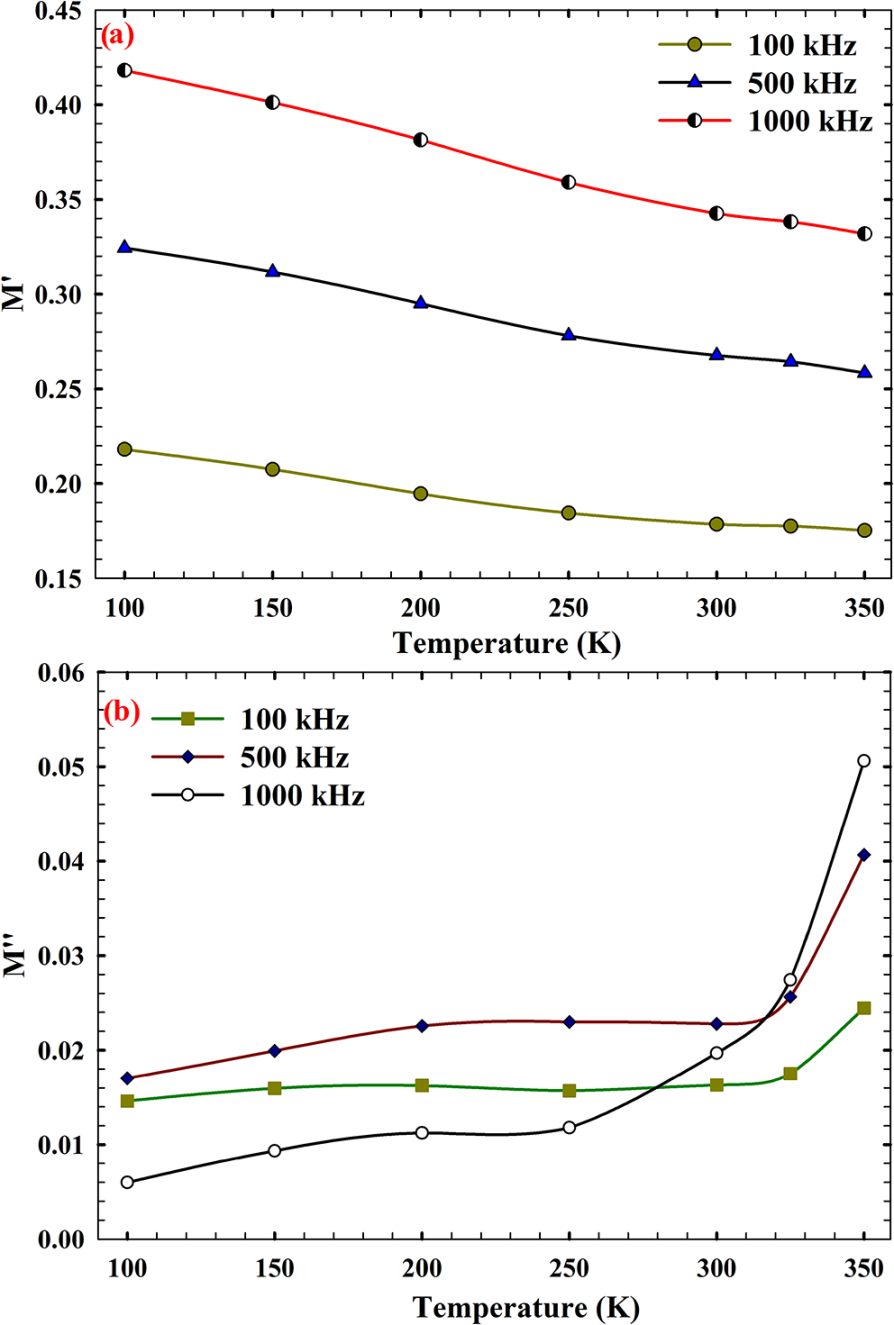
Thermal response of the electric modulus for the AlN/n-Si MIS structure: (a) real component (*M*′) and (b) imaginary component (*M*″).

The real part of the electric modulus (*M*′), as presented in Figure 9a, serves as a sensitive indicator of the material’s elastic field response and is inversely related to the dielectric permittivity. In the context of AlN/n-Si MIS structures, *M*′ provides insight into the degree of polarization suppression and the rigidity of dipolar entities under an applied AC field. The data reveal a gradual decline in *M*′ values with an increase in temperature across all frequencies, suggesting enhanced dipolar mobility and reduced field retention at elevated thermal conditions. This behavior is consistent with thermally activated relaxation, where dipoles overcome potential barriers more readily, leading to a diminished modulus response.^79^ Additionally, the frequency-dependent increase in *M′* values reflects the limited ability of slower polarization mechanisms to follow high-frequency field oscillations, thereby amplifying the elastic modulus contribution. In contrast, the imaginary part of the electric modulus (*M*″), as shown in Figure 9b, captures the dissipative component of the dielectric response and is directly associated with relaxation phenomena and energy loss mechanisms. At lower temperatures (100–250 K), *M*″ remains relatively stable, indicating minimal relaxation activity and a predominance of frozen dipolar configurations. However, beyond 300 K, a pronounced increase in *M*″ is observed, particularly at 1000 kHz, where the values rise from 0.05 to 0.10. This escalation signifies the activation of additional relaxation channels, likely involving shallow traps and interfacial dipoles that become increasingly responsive under thermal excitation. The frequency sensitivity of *M*″ further supports the presence of multiple relaxation modes, with higher frequencies favoring fast, localized processes while suppressing slower, long-range polarization.^80^

Taken together, the trends in *M′* and *M″* delineate a clear transition in the relaxation landscape of the AlN dielectric. At low temperatures and frequencies, the system exhibits a rigid polarization-dominated behavior with minimal energy dissipation. As the temperature and frequency increase, the structure evolves toward a dynamic regime characterized by enhanced dipolar reorientation, trap reconfiguration, and nonlinear relaxation. These findings not only corroborate established dielectric models but also show the unique interfacial behavior of AlN under high-frequency excitation. By integrating the modulus formalism with conductivity and permittivity insights, the study offers a comprehensive framework for evaluating the dielectric integrity and dynamic response of AlN-based MIS devices, laying the groundwork for the concluding assessment of their functional potential.

## 4 Conclusions

This study presents a comprehensive spectroscopic and impedance-based evaluation of the Au/Ti/AlN/n-Si MIS heterostructure, revealing the intricate interplay among frequency, temperature, and bias-dependent dielectric phenomena. Through systematic admittance spectroscopy and modeling, the investigation elucidates the dynamic behavior of interfacial trap states, dipolar polarization, and thermally activated conduction mechanisms across a broad operational spectrum. Capacitance–voltage and conductance–voltage analyses demonstrated that low-frequency and low-temperature regimes are dominated by trap-assisted charge dynamics and a delayed carrier response, manifesting in phenomena such as elevated dielectric loss. As the frequency increases, these effects are progressively suppressed, exposing the intrinsic dielectric behavior of the AlN interlayer and confirming its capacitive stability under high-frequency excitation. The emergence of frequency-governed transitions in *G*/ω–*V* profiles and the convergence of conductance values at elevated temperatures validate the shift from defect-mediated transport to a bulk-controlled dielectric response. Series resistance measurements revealed a strong dependence on both thermal activation and the modulation rate, with *R*_s_ values decreasing systematically as trap reconfiguration becomes kinetically unfavorable. The parallel evolution of *R*_s_, capacitance, and conductance across the examined spectrum confirms the coupled nature of dielectric relaxation and charge transport in AlN-based MIS systems. Dielectric formalism provided a deeper insight into polarization dynamics, with real permittivity increasing monotonically with temperature due to enhanced dipolar alignment and space charge polarization. Overall, the AlN/n-Si MIS structure demonstrates robust dielectric integrity, thermally modulated conduction, and a frequency-sensitive relaxation behavior. The integration of multiple analytical frameworks enabled a comprehensive understanding of the underlying physical mechanisms, positioning AlN as a promising candidate for next-generation microelectronic and optoelectronic devices operating under dynamic thermal and electrical conditions.
